# A Challenge-Based Approach to Body Weight–Supported Treadmill Training Poststroke: Protocol for a Randomized Controlled Trial

**DOI:** 10.2196/resprot.9308

**Published:** 2018-05-03

**Authors:** Avantika Naidu, David Brown, Elliot Roth

**Affiliations:** ^1^ Department of Physical Therapy and Occupational Therapy University of Alabama at Birmingham Birmingham, AL United States; ^2^ Department of Physical Medicine and Rehabilitation Northwestern University Chicago, IL United States

**Keywords:** stroke, rehabilitation, falls, walking, hemiparesis, body weight support treadmill training, balance, robotics, mobility, walking challenges

## Abstract

**Background:**

Body weight support treadmill training protocols in conjunction with other modalities are commonly used to improve poststroke balance and walking function. However, typical body weight support paradigms tend to use consistently stable balance conditions, often with handrail support and or manual assistance.

**Objective:**

In this paper, we describe our study protocol, which involved 2 unique body weight support treadmill training paradigms of similar training intensity that integrated dynamic balance challenges to help improve ambulatory function post stroke. The first paradigm emphasized walking without any handrails or manual assistance, that is, hands-free walking, and served as the control group, whereas the second paradigm incorporated practicing 9 essential challenging mobility skills, akin to environmental barriers encountered during community ambulation along with hands-free walking (ie hands-free + challenge walking).

**Methods:**

We recruited individuals with chronic poststroke hemiparesis and randomized them to either group. Participants trained for 6 weeks on a self-driven, robotic treadmill interface that provided body weight support and a safe gait-training environment. We assessed participants at pre-, mid- and post 6 weeks of intervention-training, with a 6-month follow-up. We hypothesized greater walking improvements in the hands-free + challenge walking group following training because of increased practice opportunity of essential mobility skills along with hands-free walking.

**Results:**

We assessed 77 individuals with chronic hemiparesis, and enrolled and randomized 30 individuals poststroke for our study (hands-free group=19 and hands-free + challenge walking group=20) from June 2012 to January 2015. Data collection along with 6-month follow-up continued until January 2016. Our primary outcome measure is change in comfortable walking speed from pre to post intervention for each group. We will also assess feasibility, adherence, postintervention efficacy, and changes in various exploratory secondary outcome measures. Additionally, we will also assess participant responses to a study survey, conducted at the end of training week, to gauge each group's training experiences.

**Conclusions:**

Our treadmill training paradigms, and study protocol represent advances in standardized approaches to selecting body weight support levels without the necessity for using handrails or manual assistance, while progressively providing dynamic challenges for improving poststroke ambulatory function during rehabilitation.

**Trial Registration:**

ClinicalTrials.gov NCT02787759; https://clinicaltrials.gov/ct2/show/NCT02787759 (Archived by Webcite at http://www.webcitation.org/6yJZCrIea)

## Introduction

### Background

Stroke continues to remain the leading cause of long-term neurological disability in the United States [1]. Although there is heterogeneity in the severity and level of disability post stroke, greater than 80% of all stroke survivors are likely to experience walking deficits due to hemiparesis [2]. Altered hemiparetic motor control causes balance and gait impairments, which result in asymmetric, slow (ie, 0.1 to 0.8 m/s), and inefficient walking patterns [3-5]. Such walking patterns place stroke survivors at a greater fall risk, with ambulatory stroke survivors being twice as likely to experience falls compared with elderly individuals [6,7]. Fear of falling, along with generalized deconditioning, comorbidity burden, and lack of social support and self-confidence, confines stroke survivors to sedentary lifestyles [8]. Such lifestyles limit participation in daily activities and predispose stroke survivors to secondary health conditions that negatively impact their overall quality of life [9].

### Treadmill Based Locomotor Interventions for Poststroke Rehabilitation

Not surprisingly, improving walking function is the most common rehabilitation goal stated by the majority of stroke survivors [10]. Unfortunately, most gait rehabilitation paradigms are limited in their ability to generate transferable training gains, to help improve poststroke community ambulatory function [11]. To promote motor learning and activity-dependent neuroplasticity changes during rehabilitation, increased practice of locomotor skills in different situational contexts is required [12]. However, various factors have been shown to limit context-based task practice and transferable training gains during rehabilitation, such as decreased active participation, low cardiovascular training intensities, lack of dynamic balance challenges, over-reliance on clinician manual assistance, and lack of opportunities for prolonged practice of skills applicable to real-world community ambulation [13,14]. To collectively address these factors and to promote greater opportunity for motor learning during gait rehabilitation, several studies recommend treadmill-based gait training paradigms [15], including the recent American Heart Association (AHA) scientific report for exercise training in stroke survivors [16]. However, most treadmill paradigms, especially those that integrate limb unweighting via body weight support (BWS), have had varying degrees of success over the past few decades, with some studies reporting no significant outcome differences compared with over-ground training approaches [17,18].

### Need for Challenge-Based Body Weight Support Treadmill Training Post Stroke

Most BWS treadmill paradigms also tend to use external supports (ie, safety harnesses, handrails) and/or therapist- or robot-guided movements that may limit stroke survivors from independently training at desired exercise intensities and/or the ability to challenge their dynamic balance [13,19]. In addition, the lack of context in providing training challenges to help balance confidence and walking independence for navigating through common real-world obstacles further limits training gains [20,21]. Given the variability of results with BWS treadmill training, a recent Cochrane review calls for further investigation of BWS training outcomes using task-specific paradigms of greater training intensities, without handrail support in ambulatory stroke survivors [22]. Unfortunately, safety concerns and limitations in technology restrict most BWS paradigms in their ability to provide challenging yet safe dynamic balance tasks, while training at higher intensities to help stroke survivors overcome their fear of falling and improve their walking function [16,23].

Thus, the purpose of our study was to examine 2 unique intent-driven, BWS treadmill training paradigms, of similar cardiovascular intensity that emphasizes different dynamic walking challenges encountered during community ambulation. The first paradigm involved walking without any handrail support or manual assistance (ie, hands-free [HF] walking [control group]), whereas the second paradigm incorporated practicing 9 essential challenging (C) mobility skills along with HF walking (ie, HF+C walking), relevant for navigation through common environmental obstacles/hazards. We designed both paradigms based on current neurorehabilitation [14] and AHA exercise recommendations for stroke survivors [16]. We used a novel, robotic, intent-driven, treadmill walking system [24] to provide BWS and a safe gait training environment for both groups. We were primarily interested in assessing the feasibility and impact of both treadmill training paradigms on poststroke walking performance and community ambulation capacity, respectively. We hypothesized that the HF+C group would demonstrate greater balance and functional gait improvements compared with the HF group due to increased practice opportunity of essential challenging mobility skills [25] along with HF walking.

## Methods

### Study Design

We conducted a 6-week, single-blinded, randomized, and parallel-arm study to examine the effects of 2 intent-driven, BWS treadmill training intervention groups (ie, HF training, and challenge (C) with HF (HF+C) training), on improving balance and functional walking outcomes in community-dwelling chronic stroke survivors, with mild-to-moderate hemiparetic gait impairments.

### Sample Size Estimation and Group Allocation

We used a single-factor repeated measures analysis of covariance (ANCOVA; ie, initial walking speed as a covariate) at 80% power, 2-tail level of significance of .05 (ie, *P<*.05), and an effect size of 0.4 for a gait velocity difference of 0.16 m/s (ie, minimally clinically important difference), to determine our sample size for each group. Our estimated sample size was 16 individuals per group; however, we aimed to recruit 20 participants in each group to account for attrition. Thus, our goal was to recruit a total of 40 individuals with poststroke hemiparesis, over a period of 3 years.

### Study Center

We conducted all study meetings, participant assessments, and training sessions at the University of Alabama at Birmingham (UAB) Locomotor Control and Rehabilitation Robotics Laboratory (Locolab).

### Ethics and Recruitment

We obtained study approval from the UAB Institutional Review Board (IRB protocol no: F120425008). The LocoLab program coordinator recruited study participants from the greater Birmingham area, using the UAB Stroke Registry list and an initial phone-screening form (Multimedia Appendix 1). Screened participants and their caregiver (if necessary) met with the program coordinator, who explained the study protocol in detail. Participants who provided informed consent were then scheduled for their baseline assessments.

### Initial Screening

An experienced physical therapist, blinded to the training interventions, evaluated all consented participants using our study inclusion or exclusion criteria to approve participants for study enrollment (Textboxes 1 and 2).

**Study inclusion criteria.**

1. Age 19 years and above, community-dwelling, unilateral stroke survivors
2. History of cerebrovascular accident (ie, ischemic or hemorrhagic) confirmed by computed tomography, magnetic resonance imaging, or clinical criteria
3. At least 5 months after stroke incident
4. Able to ambulate at least 14 m with/without an assistive device or the assistance of one person, with a self-selected comfortable walking speed of ≤1.0 m/s
5. Able to demonstrate receptive and expressive communication ability.
6. Primary care physician approval for exercise (obtained via the Health Insurance Portability and Accountability Act, that is, HIPPA-approved guidelines)
7. Willing to provide voluntary informed consent

**Study exclusion criteria.**

1. Presence of serious or uncontrolled cardiovascular conditions
  - Resting systolic blood pressure >180 mm Hg
  - Resting diastolic blood pressure >110 mm Hg
  - Resting heart rate >100 bpm
  - History of uncontrolled arrhythmias/angina/syncope
2. Presence of amputations and/or any severe musculoskeletal problems that restrict walking, for example:
  - Recent fractures of the lower limb
  - Open wounds/abscess
3. Use of spasticity management drug therapies for affected lower limb before participation, for example:
  - Botulinum toxin injection (<4 months earlier)
  - Phenol block injection (<12 months earlier)
  - Intrathecal baclofen or oral baclofen (within the past 30 days)
4. Any cognition involvement impairing ability to follow instructions and/or Mini-Mental State Exam Score <24
5. Past participation in any study examining the effects of long-term body weight support treadmill training in (>4 weeks of training); limb-loaded pedaling or lower extremity strengthening; or enrolled in any ongoing study that evaluates lower extremity function
6. Participant was unable to arrange for transportation to the study site for all evaluations and intervention sessions
7. Participant planned to move out of the area within 18 from the time of study enrollment

### Randomization and Stratification

**Figure 1:**
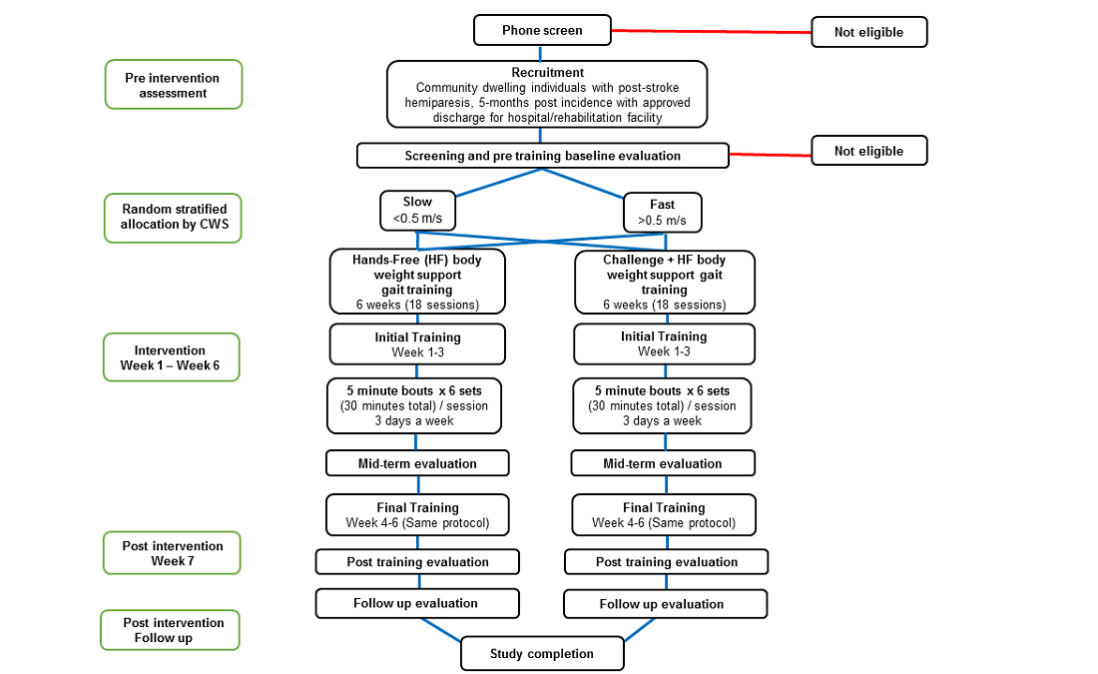
Study-flow for both paradigms from initial screening, randomization, and stratification to training (6 weeks) with follow-up at 6-months. *CWS: comfortable walking speed.

We randomized participants to each of the 2 training groups (HF or HF+C) and aimed for a 1:1 allocation ratio to minimize bias and group confounding. We also stratified participants within each group, based on their self-selected over-ground comfortable walking speed (CWS) as having mild (initial CWS<0.5 m/s) or severe (initial CWS≥0.5 m/s) locomotor impairment, using the walking speed classification by Perry et al [26] (Figure 1). We used a random number generator website [27] to generate 2 lists, of 0 and 1 sequences. We assigned participants in group 0 to the HF group and participants in group 1 to the HF+C group, using an open-ended block randomization scheme. The principal investigator (PI) assigned participants to either training group. The program coordinator gave participants their group assignment in opaque envelopes and sequentially enrolled and scheduled all training sessions and assessments, for each participant, for the duration of the study. We also blinded participants to their intervention outcomes.

### Robotic Treadmill Interface for Hands-Free Gait Training in Both Groups

**Figure 2:**
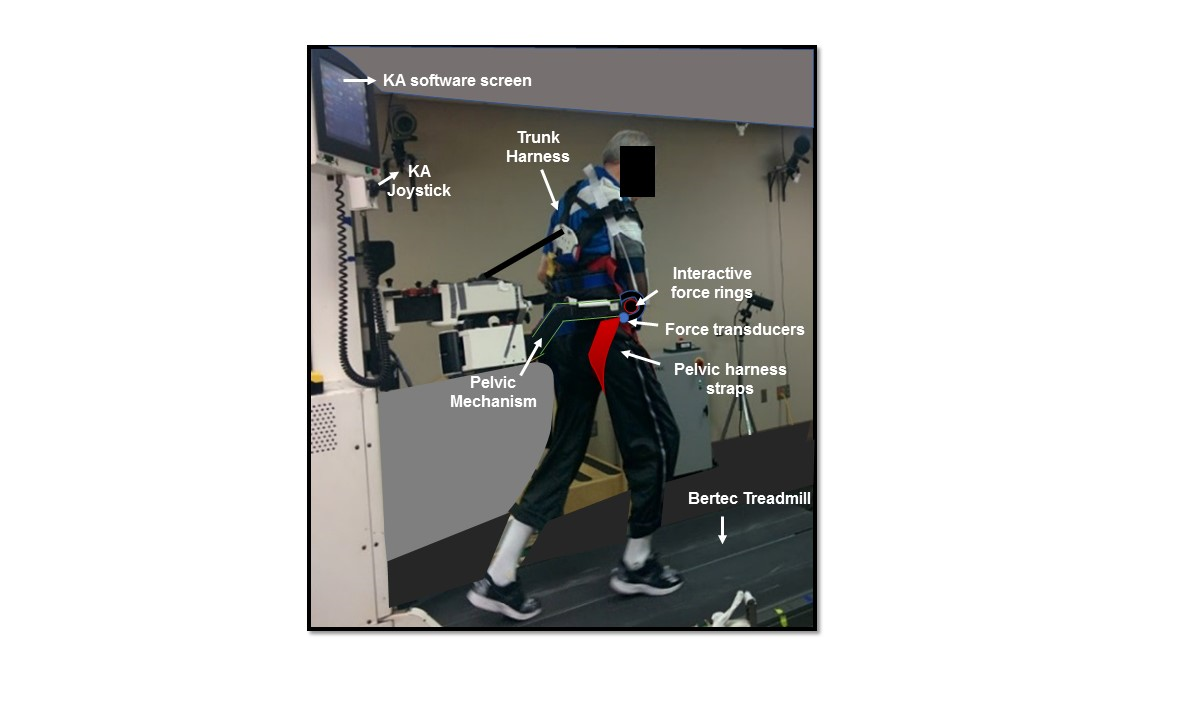
Individual poststroke walking in the KineAssist (KA) treadmill interface. The KA interface consists of a pelvic mechanism with 2 bidirectional force transducers and 2 interactive force rings attached to a pelvic harness and synced to Bertec treadmill.

Both groups trained on a novel robotic treadmill interface, which consists of a robotic-assistive device, called the KineAssist (KA; HDT Robotics, Salon Ohio, US) [28,29], synced to a Bertec treadmill [30]. The KA has been used in various studies to investigate both poststroke and nonimpaired walking biomechanics under different conditions [28,30-33]. The KA interacts with an individual walking inside it through a pelvic harness that secures their hips and waist through flexible cloth straps (Figure 2). The pelvic harness is attached to the KA’s pelvic mechanism that rests at the height of the individual’s center of mass (COM) and can provide vertical BWS (for a maximum body weight of 350 pounds and maximum height of 6 feet 5 inches). Two bidirectional force transducers at each hip enable the mechanism to sense drops in height and essentially *catch* the individual in the event of a misstep or loss of balance. This feature provides a safe environment and prevents falls during training. In addition, the force transducers and treadmill belt are paired through software to form a “force-velocity relationship”; the transducers sense the net force applied to the pelvic mechanism and send a signal to move the treadmill belt, making it an intent-driven treadmill. Thus, an individual walking inside the interface can control the speed of the treadmill and walk at their self-selected CWS (ie, intent-driven), with or without varying levels of BWS.

Unlike nonrobotic environments that use motorized treadmills combined with overhead BWS harness systems, with or without handrails/external support [17,34,35], the KA interface eliminates reliance on any external support and offers the user control over their own gait speed through the intent-driven treadmill. When the *safety-catch* feature is triggered, force rings on either arm of the pelvic mechanism (see Figure 2) allow the researcher/therapist to interactively assist the individual back to a standing position by amplifying applied forces to each ring in the vertical direction. Thus, participants learn to address their falls and stumbles inside the interface as “errors” that they can then learn to formulate strategies to prevent, as opposed to developing fears and avoiding walking behaviors that might trigger them. The pelvic mechanism also allows movement of the COM in all 3 planes through 6 degrees of freedom (DOF). Unlocking the DOFs enables the individual to explore their limits of stability, whereas locking the DOF provides external stability for those with poor balance. This feature is unique in comparison with other robotic devices, which offer limited mobility or mobility in only one plane [36]. A trunk harness also secures the individual’s trunk when they walk inside the device and prevents excessive forward lean. The KA requires a short participant setup time (5-10 min) with assistance of only 2 individuals, due to a simple computerized user interface and an easily customizable pelvic mechanism. In comparison, more sophisticated robotic treadmill gait trainer systems or nontreadmill-based robotic exoskeleton systems tend to have a long setup time and require more than 2 individuals to help set up a participant [37-39]. We have previously published details on walking biomechanics in the KA interface and its different modes in another paper [24]. However, for this study, we used 3 distinct modes of the KA treadmill interface with and without varying levels of BWS:

1. Intent-driven mode: Uses the KA’s force-velocity software relationship, which allows participants to drive the belt at their self-selected CWS.
2. Joystick mode: Enables the researcher/therapist to control (externally) the speed of the treadmill belt using a joystick controller. This mode is similar to a typical motor-driven treadmill; however, the operator is able to impose smooth or abrupt speed transitions via the KA software. We used this mode during HF+C training for speeding up and slowing down tasks.
3. KA software modifications: In either of the aforementioned modes, we used the KA software to create some of the 9 essential challenging mobility for the HF+C group. For example, using the joystick mode, we could additionally program variable speed changes, which abruptly changed the speed of the motorized treadmill belt at random intervals. In another instance, we programmed perturbations that disrupted an individual’s forward progression, while walking in the intent-driven mode.

### Training Paradigms

#### Hands-Free Body Weight Support Gait Training

This group served as our “control,” in that participants did not perform any additional essential challenging mobility skills during their 6-week training period. We felt that the inclusion of an active training control group was necessary to determine if adding essential challenging mobility skills practice to a gait training program would improve walking outcomes above and beyond improvements gained from walking practice alone. However, it is important to note that because of the safety features of our robotic device, we were able to eliminate provision of handrail support and/or manual assistance from the clinician. Although walking upright with handrail support can provide sufficient training challenge and fall safety, because of poor hemiparetic trunk control stroke, survivors are likely to adopt stooped postures by leaning forward and holding onto handrails for trunk support [40]. Such postures not only decrease training intensity and metabolic output but also minimize functional improvements [41,42]. Thus, treadmill training without handrail support can offer a more practical dynamic balance challenge that pertains to real-world independent ambulation. In addition, we did not offer participants any walking instructions (eg, how to step or correct their movements) and did not offer any passive assistance during training. Our governing principle regarding walking rehabilitation post stroke was to provide the individual with a safe environment to practice walking, solve the problem, and learn from mistakes during training. We followed AHA guidelines for exercise training [16]. Participants in this group walked for a total duration of 30 min per session, at 60% to 80% of their heart rate (HR) reserve (ie, moderate to high intensity) based on the Karvonen formula [43] with or without their prescribed BWS level (ie, between 0 and 30% support; assigned as described below). By taking advantage of the KA interface’s safety mechanism and DOF, participants in this group were able to explore their limits of stability while controlling the treadmill belt speed, and thus, train independently without assistance or external support.

#### Challenge With Hands-Free Body Weight Support Gait Training

This group served as our “experimental” group, in that participants additionally performed 9 different essential challenging (C) mobility skills, along with HF walking during their 6-week training period (Table 1). The purpose of practicing these 9 essential training challenges along with HF walking was to offer participants opportunities to navigate through common environmental hazards that they may encounter during community ambulation. This protocol was innovative, as it involved exposing stroke survivors to challenging tasks that required strong skills in anticipatory and reactive balance and functional mobility. The KA’s safety features allowed us to provide participants with this experience and to treat losses of balance or stumbles as “learning experiences,” from which participants could learn to formulate new strategies without any negative consequences. At the start of each training week, the program coordinator would randomly select and assign 3 challenges for each session using a random number generator [27]. Participants practiced training for each of the 3 skills for 30 min (10 min per skill), without handrail support at 60% to 80% of their HR reserve intensity per session. We did not have a prescribed challenge progression for each skill; however, we encouraged participants to perform each skill at a level that was challenging for them (see task difficulty, Table 1).

### Intervention Protocol for Each Training Session

This training protocol comprised 6 weeks of 18 total training sessions for both groups (summary in Table 2). Each group trained 3 times per week with alternate rest days to prevent undue fatigue.

### Participant Body Weight Support Level Determination for Each Training Session

We used a unique approach to determine BWS levels for all participants for each training session. Instead of automatically applying a specific level of BWS for all participants, we instead allowed BWS to vary per training day, depending on the participant’s fastest CWS inside the device. At the start of each session, participants walked in the self-drive mode for 5 m at 4 different levels of BWS (0%, 10%, 20%, and 30%). Some of the taller individuals were unable to use 30% because of height constraints of the KA (n=5). We calculated 10-m walk speed at each level of BWS and selected the participant’s fastest CWS using a speed difference of ≥0.08 m/s faster than 0%. The participant used this level of BWS to train for the session. This method ensured each participant’s BWS levels were individualized, unbiased, and varied according to their optimal walking speed performance. We were interested in whether participants would gradually decrease BWS over the duration of the training protocol.

### Participant Training Intensity Heart Rate Zone Determination for Each Training Session

We documented all participant’s baseline blood pressure and HR before commencement of each training session. We calculated the maximum HR for each participant using their age (ie, HR_max_=220 − age) and calculated the desired 60% to 80% training intensity using the Karvonen formula (ie, training intensity=(max HR − resting HR) × (desired %) + resting HR) based on AHA training recommendations [16]. If the participant was taking a beta-blocker, we revised this formula to use a max HR calculated as HR_max_=164 − age [44]. Thus, we individually customized the participant’s training intensity for each session. We encouraged participants to walk fast enough during training to achieve these zones; however, we also measured rating of perceived exertion (RPE; see below) to obtain a proxy measure of training intensity in the event that HRs did not reach the desired intensity. We used a GARMIN HR monitor that was strapped to each participant’s chest to record actual HR measurements for each session. We recorded HR values each minute; thus, we recorded a total of 30 HR values per training session (6 × 5-min bouts=30 min).

### Recording Training Intensity Rating of Perceived Exertion for Each Training Session

We used the Borg Scale (ie, 6 to 20) [45,46] to solicit RPE values from participants every 2 min during training. We were interested not only in participants’ general perceived training difficulty but also in which of the 9 skills would elicit the highest RPE values from participants in the HF+C group.

**Table 1:** Description of the 9 essential locomotor challenges used in training the challenge and hands-free (challenge with hands-free walking) group.

Challenge task	KineAssist interface mode	Rationale	Training practice	Task difficulty
Long stepping	KA^a^ self-drive mode	To step over common environmental hazards, for example, puddles	Using infrared laser beams, we defined a visual line on the treadmill surface in front of the participant’s feet participants instructed to take long steps, such that the heels of both feet crossed the line	If the participant was able to consistently step over the line, the distance was increased by 1-inch increments
Speeding up and slowing down	KA joystick mode	To improve the ability to speed and slow down during ambulation	The training staff the controlled the belt speed for 20 s at individual’s CWS^b^, 20 s at double their CWS, and 20 s at CWS per each minute of training	If the participant was able to successfully keep up with the fast speed, the top speed increased by 0.2 m/s
Head turns	KA self-drive mode	To simulate the need to look in different directions while walking in the community	Participants walked at their CWS. Every 10 s, staff provided instructions to turn the head either right, left, up, or down, and maintain it for 10 s	If the participant maintained walking speed with head turns, they were instructed to shake their head side-to-side or up/down for 10 s each
Variable walking speeds	KA joystick mode	To improve reactionary balance and gait speed control	KA software controlled the treadmill belt speed within a range of the participants’ CWS±0.2 m/s. Participants adapted to abrupt changes in speed	If the participant was able to successfully maintain balance and walk comfortably, speed ranges were increased by 0.2 m/s
Hurdles	KA self-drive mode	To improve ability to step over objects in the environment (eg, curb)	Participants were instructed to walk at their CWS while stepping over a hurdle positioned at height to challenge foot clearance; 5 min practice per foot	If participants consistently cleared the current hurdle height, height increased by 1-inch increments
Perturations	KA self-drive mode	To improve reactionary balance control	Participants were instructed to walk at their CWS, while experiencing abrupt disturbances (ie, brief backward accelerations) to forward progression delivered by the KA software	If participants walked through forward perturbations without experiencing disturbances (ie, missteps or backward steps), the intensity of the perturbation would be increased
Backward walking	KA self-drive mode	To improve balance control, simulate instances where stepping backward to maneuver over obstacles	Participants walked backward	If the participant successfully walked backward, they were encouraged to step faster
Walking with foam shoes	KA self-drive mode	To improve ability proprioception, to walk on uneven surfaces, and stepping height	Participants walked with foam shoes strapped to their typical footwear. Shoes ranged from 2 to 6 inches in thickness	If the participant successfully maintained their CWS, the height of the foam shoes increased from 4 to 6 inches in thickness
Narrow stepping	KA self-drive mode	To decrease reliance on external support and improve dynamic balance	Participants walked on a straight infrared while taking narrow steps at their self-selected CWS without hand support or manual assistance	If the participant successfully maintained their CWS, they were verbally encouraged to walk faster

^a^KA: KineAssist.

^b^CWS: comfortable walking speed (m/s).

**Table 2:** Summary of the hands-free and challenge with hands-free walking intervention training parameters.

Intervention	Hands-free walking	Challenge+hands-free walking
Duration	6 weeks	6 weeks
Total sessions	18 sessions	18 sessions
Weekly training	3 days a week	3 days a week
Session duration	1 hour	1 hour
Intervention duration	30 min	30 min
Training speed	Comfortable walk speed at chosen BWS^a^ level	Comfortable walk speed at chosen BWS level
Intervention goal	Perform 30 min of walking at fastest 10MWT^b^ with/without BWS as prescribed	Perform 30 min of walking at fastest 10MWT with/without BWS while performing additional walking skills
Session design	5-min bouts × sets, or as long as continuously tolerated	5-min bouts × 6 sets, or 10-min bouts × 3 sets to allow for skill changes
Session goal	Target 60% to 80% of heart rate reserve during all trials	Target 60% to 80% of heart rate reserve during all trials
Locomotor challenge	Hands-free and without manual assistance	3 new randomized locomotor total challenges per day × 3 sessions=9 per week
Instruction	Maintain heart rate in the target zone while walking	Maintain heart rate in the target zone while performing different walking skills
Physiological measures monitored	Heart rate—using heart rate, monitor each minute; rate of perceived exertion—using Borg scale every 2 min; blood pressure—pre/post	Heart rate—using heart rate monitor each minute; rate of perceived exertion—using Borg scale every 2 min; blood pressure—pre/post
Additional session measurements	Total number of steps (using step watch) and distance covered (using distance wheel)	Total number of steps (using step watch) and distance covered (using distance wheel)
Rest breaks	Every 5 min if necessary; standing breaks if heart rate exceeded zone; voluntary breaks if requested by participant (rare)	Every 5 min if necessary; standing breaks if heart rate exceeded zone; voluntary breaks if requested by participant (rare)
Training personnel	Physical therapist × 1; research assistant × 1	Physical therapist × 1; research assistant × 1
Training setting	Clinical laboratory	Clinical laboratory

^a^BWS: body weight support.

^b^10MWT: 10-meter walk test.

### Recording Total Number of Steps Taken and Distance Covered per Training Session

For both groups, we recorded the total number of steps taken per session using a step watch (Orthocare Innovations), strapped around the participant’s nonaffected ankle. We also recorded the total distance covered per training session using a Stanley distance wheel. We positioned and secured the wheel at the front of the treadmill belt and measured the distance of the moving belt while the participant walked during their training session.

### Session Duration, Approach, and Progressions

Although participants in both groups had to complete 30 min of training, each single session lasted for a total of 90 min. This included the time for baseline and post measurements/calculations (ie, blood pressure and HR), setting up the HR monitor, determination of BWS level for training, and intervention trials with/without rest breaks. Although we encouraged participants to continuously train for 30 min, we recognized that participants might not have the necessary cardiovascular endurance to continually train for 30 min. Hence, we divided each training session into six 5-min bouts. We gave participants the option to take a seated or standing rest break after completion of each 5-min bout or combine multiple bouts (ie, 10 or more continuous minutes) followed by a rest. Thus, participants could individualize their training sessions, according to their comfort and ability. We encouraged all participants, regardless of their starting point, to aggregate more bouts as they progressed with training. Research assistants, conducting the training session, verbally encouraged participants while training to maintain their CWS and finish each training bout. However, they did not provide any manual assistance or external support during training.

### Criteria for Successful Training Session Completion

Although we encouraged all participants to complete their target 30 min of training per session, we used a threshold mark of 20 min to deem a session as “complete” and include it as a data point. If a participant did not achieve the minimum of 20 min, they had to repeat the session. At the completion of each session, we documented the above-described variables and entered them into a database. The PI and program coordinator monitored this database to ensure adequate study progress and safety of all participants.

### Total Time Taken for Each Training Session Visit

Participants on an average spent 1 to 1.5 hours per training session. This included the time for evaluation and measurement of baseline parameters (blood pressure and HR), choosing appropriate BWS level, training for 30 min (including rest breaks), and final posttraining blood pressure, HR measurement, restroom breaks, and drop-off and pick-up wait. We, thus, instructed participants to keep aside 2 hours on the days they were training and up to 3 hours on the days they were assessed.

### Participant Compensation

Participants were compensated US $10 per hour for the days that they trained and were assessed.

### Participant Adherence and Missed Session Makeover

Our goal was to provide adequate rest by alternating training days with rest days per week. To support participant adherence, the program coordinator worked with participants to pick alternate training days (3 times a week) and time slots during those days that suited the participant’s schedule. However, if a participant was not able to attend their session, we rescheduled it for 1 of the 2 free days of their training week. We requested participants to keep at least 2 hours aside for training on the days that they committed to come, and up to 3 hours aside for the days they would be assessed. We instructed participants that it was critical that they did not miss any training sessions and enrolled participants only after they had finished any travel obligations that would have interfered with their training. In addition, the program coordinator would also call and remind participants, a day before their training session, to come for training. If participants did not have a personal means of transport to come to the LocoLab for training, we arranged for alternate local public transport options, for example, local government run bus/van service. We also limited participants from rescheduling and extend their training sessions to a maximum of 7 consecutive weeks, to complete their 18 sessions, taking into account any rescheduled sessions because of personal commitments and/or national holidays. Participants were allowed a total of 5 rescheduled sessions.

### Minimizing Variability in Application of Procedures

We ensured that a minimum of 2 research staff members, one being a physical therapist, trained every participant during each training session. In total, we had 10 different research staff members, including 3 physical therapists, who regularly rotated and conducted all the training sessions to minimize expectation bias. The PI oversaw all training sessions and ensured strict adherence to all training protocols for both groups. The program coordinator and PI reviewed the progress of both training groups weekly and checked if all data were correctly documented. We ensured that no cointervention contamination occurred, by asking participants to refrain from attending any active lower limb physical therapy programs or participating in any walking intervention studies outside of our study.

### Reporting of Adverse Events

We defined an adverse event as an event that occurred during or after the training session when the participant was at the training site and trained staff members to report any adverse event pertaining to:

1. Fall to the ground (defined as an unintentional loss of balance)
2. Any symptoms of angina or myocardial infarction
3. Any musculoskeletal injury during/after session training
4. New stroke or transient ischemic attack
5. Hospitalization for any cause
6. Death due to any cause.

Participants were also encouraged to report any symptoms (pain, soreness, numbness, etc) or signs of injury (inflammation, blisters, etc) that they experienced following training on returning home.

### Standard Precautions

We used the same standard precautions for both training groups and modified them for each individual participant, after evaluation and recommendation by the PI. These included the following:

1. Decrease in exercise intensity for systolic blood pressure greater than 200 mm Hg or diastolic blood pressure greater than 100 mm Hg.
2. Decrease in exercise intensity, if HR was greater than 75%.
3. Pause in training on observation of dyspnea or if blood pressure dropped below resting pressure.
4. Pause in training if participant reported symptoms of light-headedness.

### Assessments

We used various functional mobility assessments at different time points - pretraining (baseline), midterm, posttraining (final), and 6-month follow-up, conducted by the physical therapist on our study at the LocoLAB. We used the 10-meter walk test to measure participants’ CWS and fast walking speed (FWS) [47], and the 6-min walk test (6MWT) [48] to measure walking capacity using an 85-feet oval walkway. At baseline, participant’s hemiparetic severity and ambulation category were classified using the lower extremity Fugl-Meyer, and functional ambulation category scale [49], respectively. We also used the Mini-Mental Scare Examination as a screening tool for participants’ cognitive function (>24) [50]. We also used the Berg-Balance Scale (BBS) [51] and Dynamic Gait Index [52] to measure participants’ balance function. We used the Activities-specific Balance Confidence (ABC) scale [53] to evaluate participants perceived balance function during activities of daily living, and the Geriatric Depression Scale (GDS) [54] and Stroke Impact Scale (SIS) [55] to assess participant’s mental function and perceived impact of poststroke disability on their quality of life, respectively. Participants were assessed at baseline, midterm, final (after 6 weeks), and at 6-month follow-up. Table 3 describes the assessments performed during these periods.

**Table 3:** Timeline for assessments and collection of outcome variables at various study stages.

Baseline		Midterm	Final	6-month follow-up
Comfortable walk speed (CWS)		CWS	CWS	CWS
**Fast walk speed (FWS) using**		FWS	FWS	FWS
	10-meter walk test (10MWT)	6MWT	6MWT	6MWT
	6-min walk test (6MWT)	BBS	BBS	BBS
Fugl-Meyer lower extremity score		GDS	GDS	GDS
Functional Ambulation Category		—	DGI	DGI
Berg Balance Scale (BBS)		—	ABC	ABC
Dynamic Gait Index (DGI)		—	SIS	SIS
Geriatric Depression Scale (GDS)		—	—	—
Stroke Impact Scale (SIS)		—	—	—
Activities-Specific Balance Confidence (ABC)		—	—	—
Mini-Mental State Examination (MMS)		—	—	—

### Primary Outcome Measure

As change in over-ground CWS is an important, valid, sensitive, and reliable measure of poststroke recovery and walking function [56], we chose difference in CWS between groups, from pre- to posttraining as our primary outcome measure [57,58]. On the basis of our power analysis, we planned to include baseline-walking speed as a covariate, if it was significantly related to CWS at pre- and posttraining.

### Secondary Outcome Measure

We plan to report descriptive data on the following secondary exploratory outcome measures: FWS, 6MWT, BBS, GDS, SIS, and ABC scores, with mean and SDs. However, we will not include these variables in our main analysis, as we are not appropriately powered to include them.

### Subgroup Analysis

In addition to these main assessment measures, we also recorded BWS levels, HR, and RPE exertion changes, total number of steps, and distance covered during each session, for participants in both training groups. Descriptive data analysis along with subgroups comparative analysis will be performed on these variables, from pre- to postintervention and on a week-by-week basis, and will be reported in another manuscript. We will also assess feasibility and compliance of each group to either intervention, along with ability to maintain target HR intensity during training. These subgroup analyses will help in better understanding the impact of training on functional walking outcomes and impact on community ambulatory function.

### Survey Data Analysis

Participants in both groups also completed our custom-designed study survey at the end of each training week. The survey consisted of questions on the 9 essential challenging mobility skills that we identified using the research criteria specified by Patla et al [25]. The survey questionnaire consisted of the following 3 subparts: (1) identify which of the 9 essential challenging mobility skills you have difficulty with in your daily life (using yes/no responses); (2) rank in order of importance (1-9), which of the 9 essential mobility skills is most important for you in improving walking function; and (3) respond to specific questions on your training experiences (using a Likert scale 1-7; see Multimedia Appendix 2). We will assess survey data responses for change in responses, ranking of task difficulty, and change in Likert scale scores, respectively.

### Criteria for Data Analysis

We will only be considering those participants in our final analysis, as a data point, if they completed their first week of training. Participants will not be included in our final analysis if they were unable to complete the first week of training, for reasons such as personal time limitations, conflicting time commitments, family crises, and personal psychological factors such as depression and medical procedures.

## Results

### Total Participants Enrolled in Our Study

We assessed 77 individuals with chronic mild to moderate hemiparesis, and excluded 38 individuals who did not meet our inclusion criteria, from June 2012 to January 2015. We enrolled and randomized a total of 39 individuals post stroke for our study, with 19 participants in the HF group and 20 participants in the HF+C group. Data collection along with 6-month follow-up continued until January 2016. Detailed results of this study will be presented in 2 subsequent manuscripts.

### Proposed Statistical Methods

We will assess normality and homogeneity of data for all outcome measures in both groups. If possible, we plan to conduct an intention-to-treat analysis. We will compare our primary measure in both groups from pre- to postassessment using an ANCOVA at a significance level of *P*<.05, with the covariate being baseline CWS, if significantly related to change in CWS from pre- to posttraining. As all our secondary outcome measures are exploratory in nature, we will use a repeated measures design for comparing changes from baseline, midterm, final assessment, and at 6-month follow-up, at a significance level of *P*<.05, with post hoc analysis. For our survey data, we will compare changes between the first and last training sessions using Spearman correlation for the yes/no responses question, a Wilcoxon rank-sum test for the rank order question, and chi-square analysis for Likert scale responses. A subsequent manuscript will have detailed description of our data analysis methods and statistical tests.

## Discussion

Locomotor disability post stroke continues to impede stroke survivors from engaging in active community participation and negatively impacts their quality of life. Given the prioritization of improvement in walking function by stroke survivors and the 2014 AHA exercise training recommendation report, it is all the more vital that poststroke gait interventions incorporate task-specific essential challenging mobility skills, akin to real-world scenarios, along with training at higher cardiovascular intensities to improve functional gains [59]. Through our study, we explored 2 unique gait interventions, ie, HF walking and HF+C walking, using a novel and safe gait training environment to investigate functional ambulation capacity after 6 weeks of training. Our study results will yield important insight on whether individuals post stroke can train at higher intensities, especially while walking without any external handrail support or passive manual assistance at their preferred self-selected BWS level (HF walking) or when practicing essential challenging mobility skills with HF walking (HF+C walking). Although our primary outcome measure is change over-ground CWS, we have also collected various secondary exploratory measures and survey data for both training paradigms. As our secondary measures are most commonly used in clinical settings to assess functional ambulatory capacity, we hope these variables will inform future poststroke studies that plan to use similar gait training paradigms. Our main study outcomes and week-by-week training analysis are being presented in 2 separate manuscripts, respectively. We hope that the results of this study will help in better informing clinicians and researchers on how real-world balance challenges can be incorporated to improve the selection of treadmill training protocols to improve functional walking capacity for individuals post stroke.

## Appendix

Multimedia Appendix 1 Participant Phone Screen Form.

Multimedia Appendix 2 Weekly Community Walking Survey.

## Abbreviations

6MT: 6-min walk test
10MWT: 10-meter walk test
ABC: Activities-Specific Balance Confidence scale
AHA: American Heart Association
ANCOVA: analysis of covariance
BBS: Berg Balance Scale
BWS: Body weight support
COM: center of mass
CWS: comfortable walking speed
DOF: degrees of freedom
FWS: fast walking speed
GDS: Geriatric Depression Scale
HF: hands-free
HF+C: challenge and HF walking
HR: heart rate
KA: KineAssist
KA interface: KineAssist treadmill interface
PI: principal investigator
RPE: rate of perceived exertion
SIS: Stroke Impact Scale
UAB: University of Alabama at Birmingham
