# Supplementary material for: A Challenge-Based Approach to Body Weight–Supported Treadmill Training Poststroke: Protocol for a Randomized Controlled Trial
Source: JMIR Res Protoc. 2018 May 3;7(5):e118. doi: 10.2196/resprot.9308 (PMC5958283; doi:10.2196/resprot.9308)
Supplement: Multimedia Appendix 1 [file resprot_v7i5e118_app1.pdf]

Appendix 1

Phone Screen Form

Before I begin, I'd like to tell you that I will be collecting information about you during this phone call. Your information will only be seen by researchers at UAB. We try to make sure that the information we collect from you is kept private and used only for the research study we are discussing. If you do not agree to continue the phone call, it will not affect your care at UAB. Study records that can identify you will be kept confidential by keeping your records in a password protected computer and the paper files will be kept in a locked file cabinet at UAB. All data will be stored with a study code allowing only research staff to review.

Interviewer Name ____________________ Interviewee Name: ______________ Date___________

Subject ID _______________________________DOB:_____________________ Male Female

Physician: ______________________ Phone Number:________________________________

1. When was your stroke? ____________ CVA: Right Left Affected Side: Right Left
2. Was this your first stroke? ____________ CVA confirmed on: MRI CT Scan
3. Are you able to walk? Yes No If yes, how far? ________________________________
   a) Do you use an assistive device when you walk? Yes No

      (ie: SPC, 2ww, 4ww, quad cane, etc.) If yes, what kind? ______________________________
   b) Do you wear a brace on your weak leg when you walk? Yes No

      If yes, all the time? _____________________________
   c) Do you use a wheelchair? Yes No

      If yes, when? __________________________________

d) Are you able to straighten/bend your weak knee and move your weak ankle?
Yes No

If yes, how much? ________________________________

4. Did you have any walking difficulties prior to your stroke? Yes No

5. Do you have any serious medical conditions?
Yes No

(obtain list of current medications) If yes, what are they? ____________________________

6. Do you have significant pain that interferes with your ability to go about your daily activities? Yes/No

7. Are you currently participating in any type of stroke therapy?
Yes No

PT_______________ OT_______________ Other_______________

8. Are you currently in any other research projects? Yes No

If yes, what kind are they? ________________________

9. Are you currently taking any medicine (baclofen) or had any shots (botox) to make your arm or leg less stiff?
Yes No

(Note: Differentiate from medication taken for joint stiffness/arthritis)

10. Do you have resources for transportation to and from our facility?
Yes No

11. Do you have a caregiver available? Yes No

If yes, who is your caregiver? ______________________________

12. Can I or my fellow research colleagues contact you for other studies involving stroke rehabilitation? Yes No

Comments:
Surgerires____

____
____

After going over the phone screen, ask:

Do you have any questions?

____
____
____
____
