# Supplementary material for: A Challenge-Based Approach to Body Weight–Supported Treadmill Training Poststroke: Protocol for a Randomized Controlled Trial
Source: JMIR Res Protoc. 2018 May 3;7(5):e118. doi: 10.2196/resprot.9308 (PMC5958283; doi:10.2196/resprot.9308)
Supplement: Multimedia Appendix 2 [file resprot_v7i5e118_app2.pdf]

Appendix 2

Weekly Community-Walking Survey

---

1. Patient's Study ID: _______ - _________________ - __________

Study ID   Seq. Patient Number   Last Name (1st 2 letters)

2. Evaluation ___Baseline ___Mid-treatment ___Post-treatment ___6-month follow-up

3. Evaluation Date: ______/______/___________

Month   Day   Year

4. Evaluator's Initials: ________

First/Last

---

1. **Which of these tasks do you have trouble doing in your daily life?**

   a) Speeding up and slowing down suddenly while walking? Y/ N
   b) Taking long steps? Y/ N
   c) Stepping onto a slippery surface (icy or wet) Y/ N
   d) Stepping on uneven surfaces? Y/ N
   e) Taking narrow steps? Y/ N
   f) Being bumped into by someone or something from the front? Y/ N
   g) Being bumped into by someone or something from the back? Y/ N
   h) Walking sideways? Y/ N
   i) Stepping over a tall object? Y/ N

2. **Rank from 1 to 9 which of these tasks you would like to improve upon:**

______Speeding up and slowing down suddenly while walking?

______Taking long steps?

______Stepping onto a slippery surface (icy or wet)?

______Stepping on uneven surfaces?

______Taking narrow steps?

______Being bumped into by someone or something from the front?

______Being bumped into by someone or something from the back?

______Walking sideways?

______Stepping over a tall object?

3. For each of the following statements, please indicate how true it is for you, using the following scale:

1	2	3	4	5	6	7
**Not at all true**			**somewhat true**			**very true**

Please write the number corresponding to your selection in the blank before each question. Please answer these questions in relation to your recent physical therapy experiences here at this lab.

1. ___ I enjoyed doing this activity very much
2. ___ I believe doing this activity could be beneficial to me.
3. ___ I thought this was a boring activity.
4. ___ After working at this activity for a while, I felt pretty competent.
5. ___ I would describe this activity as very interesting.
6. ___ I didn’t try very hard to do well at this activity.
7. ___ While I was doing this activity, I was thinking about how much I enjoyed it.
8. ___ I did not feel nervous at all while doing this.
9. ___ I think I am pretty good at this activity.
10. ___ I felt pressured while doing these.
11. ___ I didn’t put much energy into this.
12. ___ I think I pretty well at this activity, compared to other participants.
13. ___ I put a lot of effort into this.
14. ___ I would be willing to do this again because it has some value to me.

15. ___ This was an activity that I couldn’t do very well.
16. ___ I felt very tense while doing this activity.
17. ___ I tried very hard on this activity.
18. ___ It was important to me to do well at this task.
19. ___ I am satisfied with my performance at this task.
20. ___ I thought this activity was quite enjoyable.
21. ___ I was very relaxed in doing these.
22. ___ I was anxious while working on this task/anxious to finish.
23. ___ This activity did not hold my attention at all.
24. ___ I believe this activity could be of some value to me.
25. ___ This activity was fun to do.
26. ___ I think that doing this activity is useful or rehabilitation.
27. ___ I think this is important to do because it can help me to recover.
28. ___ I think doing this activity could help me to recover.
29. ___ I think this is an important activity.
30. ___ I pretty skilled at this activity.
